# “Cohesiveness of Hyaluronic Acid Fillers”: Evaluation Using Multiple Cohesion Tests

**DOI:** 10.1055/a-2234-1019

**Published:** 2024-01-24

**Authors:** Kyun Tae Kim, Won Lee, Eun-Jung Yang

**Affiliations:** 1 Yonsei Dain Plastic Surgery Clinic, Seoul, South Korea; 2 Scientific Faculty of the Minimal Invasive Plastic Surgery Association, Seoul, South Korea; 3 Yonsei E1 Plastic Surgery Clinic, Anyang, South Korea; 4 Department of Plastic and Reconstructive Surgery, Yonsei University College of Medicine, Seoul, South Korea

**Keywords:** soft tissue filler, hyaluronic acid filler, cohesion, filler property

## Abstract

**Background** Hyaluronic acid fillers can be manufactured using various processes. They have multiple properties, including their concentration, degree of modification, and rheological data. Cohesion is one such property to evaluate gel integrity; however, there is no standardized method for calculating this parameter. This study aimed to evaluate different tests for calculating hyaluronic acid cohesion and discuss the importance of hyaluronic acid cohesion as a consideration when selecting fillers.

**Methods** The cohesion levels of five different hyaluronic acid fillers with different rheological properties were evaluated and compared using the drop weight, compression, tack, and dispersion time tests.

**Results** The cohesion tests yielded different results in the samples. Samples 2 and 4 showed approximately two times the number of droplets when compared with Sample 5 in drop weight test. Samples 1, 2, 3, and 4 were superior to Sample 5 in tack test. Samples 1, 2, and 3 showed cohesive appearances at 95 seconds in most cases in dispersion test. Rheological test results did not reflect the measures of cohesion.

**Conclusion** Although there are no definite standardized tests to evaluate the cohesion of hyaluronic acid fillers, our proposed tests showed similar results for different hyaluronic acid filler products. Further studies are needed to evaluate the cohesion of hyaluronic acid fillers and determine the clinical use of this distinguishing characteristic for clinicians selecting the product of choice.

Level of evidence statement: These data are Level IV evidence.

## Introduction

The use of soft tissue fillers is one of the most commonly used aesthetic procedures for facial rejuvenation. Among the soft tissue filler, it is well-known that hyaluronic acid filler is most commonly used. Although filler injections are easy to perform, they have complications such as vascular and nonvascular. Nonvascular complications include infection, migration, delayed inflammatory reaction, unsatisfied nodule, and these complications are sometimes related with hyaluronic acid filler properties. Thus, it is essential to understand hyaluronic acid filler properties such as rheologic data. A hyaluronic acid filler is a viscoelastic material that exhibits both viscous and elastic behavior when undergoing shear deformation. 1 Rheological data can be used for understanding the properties of a hyaluronic acid filler and for appropriate clinical application. 2 However, rheological properties only encompass one aspect of the filler and cannot sufficiently predict filler performance. 3 Although the elastic modulus, G′, represents a useful and consistent parameter for product differentiation, there are still many potentially different properties that impact product characteristics. 4

Cohesion is defined as the internal adhesion forces holding together individual cross-linked hyaluronic acid units in a hyaluronic acid gel deposit. 5 Cohesion is a parameter often mentioned in the literature on dermal fillers, which contribute to tissue support and measurement of gel integrity. High cohesivity helps fillers maintain vertical projection when stress is applied by soft tissues. 1 However, gel cohesion is not scientifically recognized as an appropriate property for product comparison, owing to the lack of a standardized measurement technique. 4 6 Nonetheless, cohesion is an important characteristic of a hyaluronic acid filler and should be evaluated separately from other physicochemical parameters such as rheology. 7

In this study, we aimed to evaluate different tests for calculating hyaluronic acid cohesion and discuss the importance of hyaluronic acid cohesion. This is the first study to examine cohesion measures, exploring their importance between fillers from other manufacturers developed with clinically similar indications.

## Methods

### Materials

Five different hyaluronic acid fillers were selected and labeled as Samples 1 to 5 for the four kinds of cohesion tests. The chosen fillers are recommended to be used at nasolabial fold correction by manufacturing company.

The filler samples were labeled as follows:

Sample 1: EPTQ lidocaine S300 (YL21014, Jetema, Seoul, South Korea)
Sample 2: Chaeum Premium No. 2 (BLC21000A, HUGEL, Seoul, South Korea)
Sample 3: Neuramis Deep lidocaine (F121022A, Medytox, Seoul, South Korea)
Sample 4: Elravie Premier deep line (V04113, Humedix, Seoul, South Korea)
Sample 5: Yvoire Volume (IVP21008, LG, South Korea).

### Rheological Tests

Rheologic tests were performed on all five samples for comparing the general rheologic data with results from the cohesion tests. Because rheologic data are well-known and often used for clinical usage. The elastic modulus, G′, was measured in a frequency sweep within the linear viscoelastic range determined by strain sweep. The properties were measured from 1 to 0.1 Hz and data were compared. G″ complex viscosity, and tan delta were also evaluated.

**Fig. 1:**
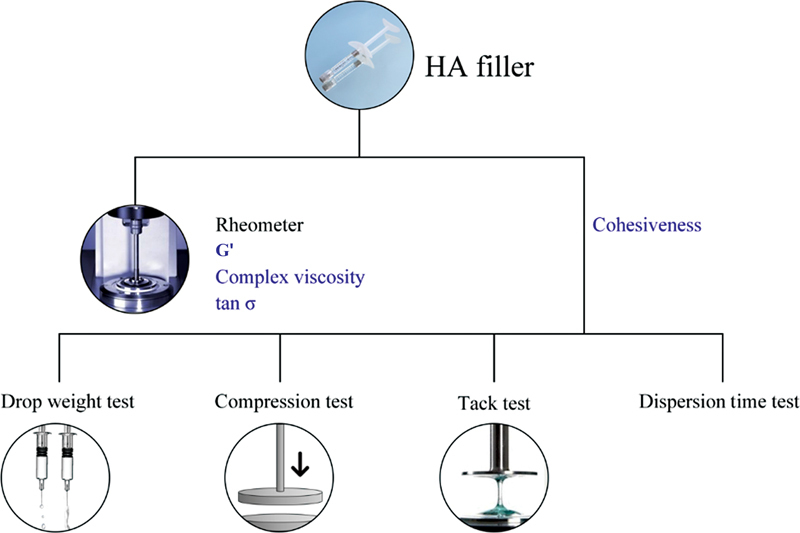
Schematic diagram of the experiment. Rheological test results were compared with cohesion tests.

The injection force was measured mechanically (MultiTest2.5-dv and AFG, Mecmesin LTD, Horsham, United Kingdom). Each filler was injected under the same conditions using 27G needles (Taechang, Seoul, South Korea). Rheological test results are compared with cohesion tests ( Fig. 1 ).

### Tests for Cohesion

Four tests for determining cohesion were performed on all five filler samples.

**Fig. 2:**
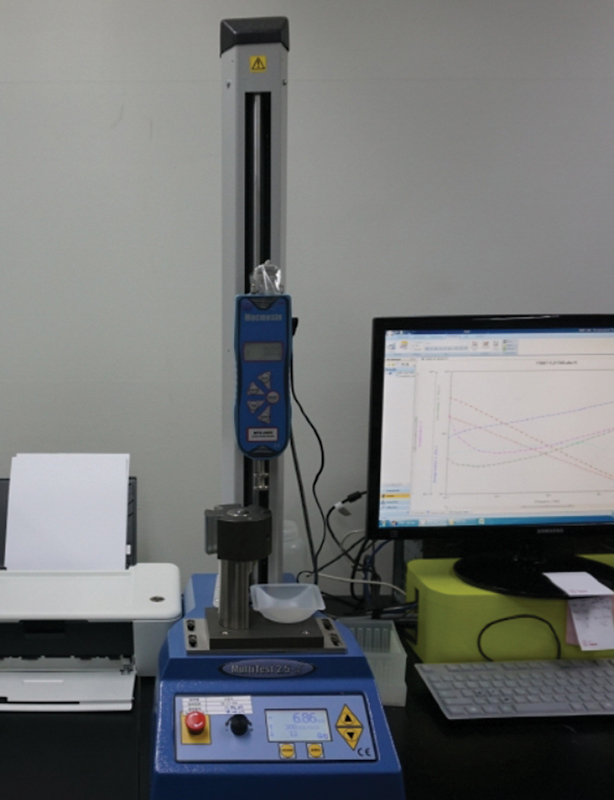
Drop weight test. Hyaluronic acid filler; 20 droplets were tested for weight.

**Fig. 3:**
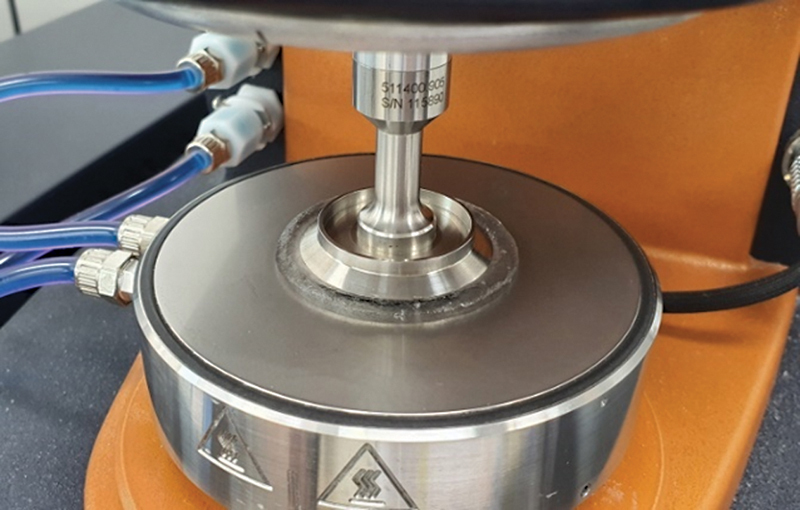
Compression test. Hyaluronic acid fillers were put at the center of plate of rheometer.

1. **Drop weight test** ( Fig. 2 ): A hyaluronic acid filler with prefilled syringe was placed in the test machine (MultiTest 2.5-dv and AFG, Mecmesin LTD, Horsham, United Kingdom). Test machine is used for extrusion force analysis but also can be used for drop weight test. When filler is placed at the test machine, extrusion was performed at a constant speed of 3 mm/min. A 21G needle was attached to the filler samples and extrusion started. Because hyaluronic acid filler is viscoelastic material, droplet is showed by automatic machine and one droplet of each sample was analyzed. When a constant force was achieved, 20 drops were collected, and the average weight was calculated.
2. **Compression test** ( Fig. 3 ): A hyaluronic acid filler was placed on the plate of the rheometer (Discovery HR2, TA, Korea). A parallel plate diameter of 40 mm and temperature of 25°C were used. The upper plate was moved downward to compress the filler specimen, and the forces were calculated. One milliliter each of the five samples was centered on the lower plate. The samples were allowed to relax for 118.448 seconds.
3. **Tack test** : A hyaluronic acid filler was placed on the plate of the rheometer. After the compression test, the upper plate was moved upward. When the compressed hyaluronic acid filler was separated, adhesion force was calculated.
4. **Dispersion** : Time test (also known as the Gavard–Sundaram Cohesivity Scale 6 ): All five hyaluronic acid filler samples were stained by adding 30 μL solution of toluidine blue and mixed for 3 minutes. Colored hyaluronic acid gel specimens were extruded under standardized conditions into sterile water chamber and stirred at a constant rate. A magnetic stirring bar, 25-mm long was used at a constant rate of 170 rpm. Dispersion was evaluated with serial photographs captured at 15, 70, and 95 seconds. The cohesivity scale was used for calculation. Each photograph was graded by two evaluators on a scale of fully dispersed (1), partially dispersed (2), partially cohesive (4), and fully cohesive (5).

## Results

### Rheological Test

**Table 1:** Rheological data of hyaluronic acid fillers tested

Sample	Concentration (mg/mL)	Injection force (N)	Rheologic data at 0.1 Hz			
			G′ (Pa)	G″ (Pa)	Complex viscosity (Pa·s)	Tan delta
1	20	12.134	156.06	27.50	252.20	0.176
2	23	15.768	166.45	30.97	269.46	0.186
3	20	7.546	129.75	37.38	214.90	0.288
4	24	11.309	151.30	30.38	245.60	0.201
5	22	8.298	381.19	54.72	612.91	0.144

The fillers used in this study were chosen and were compared with each other with the same clinical indication (recommended to use at nasolabial fold correction) and their elastic modulus was evaluated from 129 to 381 Pa at 1 Hz ( Table 1 ). Sample 5 showed highest elastic modulus and this parameter is usually related to elasticity. Complex viscosity varies from 214.90 to 612.91 Pa·s, and Sample 5 showed highest complex viscosity. Tan delta results were from 0.144 to 0.288. Thus, Sample 3 showed more solid-like filler than other filler samples.

### Drop Test

**Table 2:** Drop test results of the five hyaluronic acid fillers

Drop No.	Sample 1	Sample 2	Sample 3	Sample 4	Sample 5
1	16.7	20.9	19.3	19.9	11.9
2	18	22.6	18.7	21.6	12.2
3	17.1	22.4	17.3	23.6	11.3
4	16	24.5	17.1	20.7	11.9
5	17.6	22.9	18	22	11.4
6	16.4	21.1	15.7	22.8	11.1
7	16.9	22.8	17.2	21.2	13.3
8	16.8	21.5	17.3	21.3	11.3
9	16.8	21.7	16.6	22.3	10.8
10	16.4	22	18.7	19.8	11
11	17.6	20.7	16.5	22.5	9.3
12	15.4	21.5	17.8	20.3	10.1
13	16.8	22.6	17.8	17.3	8.1
14	17.3	22	17.1	23.5	11.4
15	16	24.2	17.4	24.6	12.2
16	16.3	21.3	17	23.1	11.6
17	16	23.3	16.6	20.1	11.4
18	17.6	21.1	18.3	22	10.3
19	15.5	21.1	16.8	20.3	11.8
20	16.8	20.3	16.7	19.6	12.3
Mean ± SD	16.7 ± 0.7	22.0 ± 1.1	17.4 ± 0.9	21.4 ± 1.7	11.2 ± 1.1

Abbreviation: SD, standard deviation.

Samples 2 and 4 showed approximately two times the number of droplets when compared with Sample 5 ( Table 2 ). Thus, one can estimate that Samples 2 and 4 had twice the internal adhesion force of Sample 5.

### Compression Test

**Table 3:** Compression test results

Sample No.	Step time (seconds)	Normal stress (Pa)	Axial force (N)	Gap (μm)
1	118.448	4327.81	2.72	910.789
2	118.448	5064.28	3.18	910.902
3	118.448	2681.69	1.68	910.754
4	118.448	5241.34	3.29	910.684
5	118.448	2115.96	1.33	910.941

The compression and tack tests were performed using a rheometer. Compression test results showed that Samples 3 and 5 were easier to compress ( Table 3 ). Clinically, compression force can also increase when there are large hyaluronic acid particles.

### Tack Test

**Table 4:** Tack test results

Sample No.	Step time (seconds)	Gap (μm)	Axial force (N)
1	0.26	523.88	−11.68
2	0.24	522.79	−11.03
3	0.23	519.23	−9.38
4	0.25	523.82	−10.90
5	0.15	512.35	−5.82

Conversely, tack test results were similar those of the drop weight test, which showed that Samples 1, 2, 3, and 4 were superior to Sample 5 ( Table 4 ). However, this can also be affected by the stickiness of the hyaluronic acid filler and the rheometer plate. Viscosity of the hyaluronic acid filler can also be attributed to this outcome.

### Dispersion Time Test

**Fig. 4:**
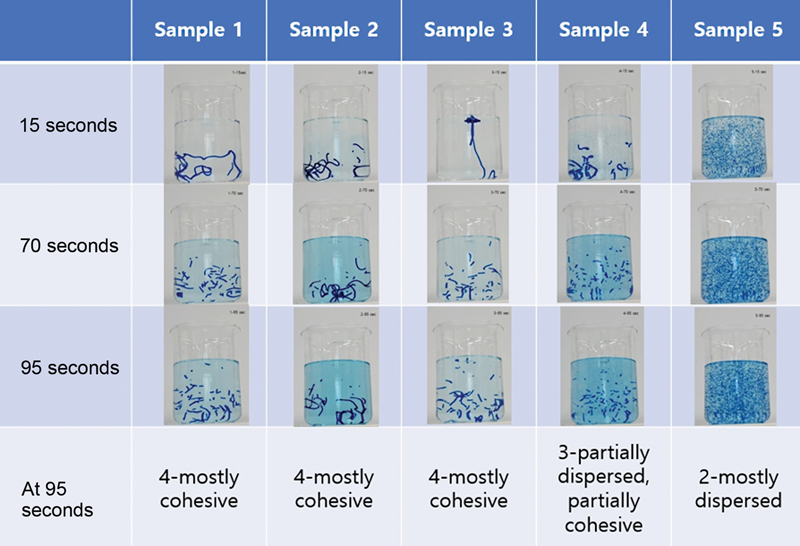
Dispersion time test result. Samples 1, 2, and 3 showed cohesive appearances at 95 seconds.

Samples 1, 2, and 3 showed cohesive appearances at 95 seconds in most cases ( Fig. 4 ).

## Discussion

In our study, five different hyaluronic acid fillers from other manufacturers developed with clinically similar indications were evaluated using four cohesion tests.

In Table 1 , in the case of Sample 5, also known as biphasic filler, the hyaluronic acid concentration is also a median value compared with the concentration of other fillers, and the viscosity (G″) and elasticity (G′) on the rheological test are very high and relatively high, respectively. In addition, the tan delta value is 0.144, which is the lowest among the five samples, hence, the elasticity is very high and the complex viscosity is the highest. When predicting filler properties with only existing rheological data, the filler in Sample 5 shows high elasticity and excellent tissue-lifting ability. However, these results were not found in clinical use, the effectiveness of the treatment area decreased with time faster than clinically expected, failing to maintain the original shape. This indicates that the objective rheological data alone do not fully describe the clinical performance and characteristics of the filler and suggest that cohesion properties should be considered as well.

The result of the drop weight test shows the average value obtained by measuring the amount of one drop several times when the hyaluronic acid gel is dropped by gravity by moving the syringe at the same speed. If long strands are observed under magnification, the gel is deemed cohesive. If the gel breaks down into particles, it is noncohesive. The definition of cohesion refers to the property of not separating between molecules or particles, and the amount of drops formed when a gel particle is pulled down by the same force gravity fits the definition very well and reflects the characteristics of cohesion well. The drop weight test is known for yielding results that highly correlate with those of the perceived cohesion test. 7 The perceived cohesion test evaluates how sticky the filler is based on finger perception. Since this is a subjective method, it was not included in our study.

These results suggest that these gels do not remain monophasic and will not remain evenly distributed after injection, as they form into clumps of material. This, however, may not necessarily be an issue, depending on the desired aesthetic outcome. Products with higher average drop weights correlated with lower G′ values were deemed more cohesive. When the filler is applied to a tissue, one can estimate that Samples 2 and 4 will aggregate better than Sample 5 and be easier to mold after filler injection. Molding is one of the easiest techniques for making filler result naturally. For example, when hyaluronic acid filler is injected into forehead area, molding technique is necessary for showing natural forehead contour. Otherwise the results might not be satisfactory because of irregularity. Thus, it is important to know whether the filler is highly cohesive or highly elastic and decide the appropriate application. For forehead area, augmentation is important but smooth contour without irregularity is more important. For nose augmentation, it is absolutely important to augmentation. Cohesion is also important for filler migration. When there is not enough cohesion, filler might migrate. Most of all, cohesiveness is related with maintenance of injected filler. Maintaining the initial height of injected filler is one of the important factors for choosing proper filler.

The compression test results refer to the resistance before and after the gel causes deformation on two plates acting vertically. Therefore, in general, it is easy to think that the compression test results will be high in fillers with strong complex viscosity, evenly reflecting viscosity and elasticity. However, considering that the compression force was low in the case of Sample 5, which showed a very strong level of complex viscosity, it is difficult to say that the filler characteristics are clearly shown with the rheological data alone.

Since the tack test is a result of reflecting an adhesion force that does not want to be separated between the two plates, it may be considered appropriate to think that the filler in Sample 1, whose value is measured the highest, has a high cohesive force. However, the high tack test results contrast with the low results of the filler in Sample 1 in the drop weight test. Therefore, if coherence is a value that must be judged comprehensively through several kinds of cohesion tests, it may not be appropriate to evaluate it with only one kind of cohesion test result.

The characteristics of fillers should be identified and used by comparing various physiochemical and rheological data representing the characteristics of fillers with various methods of understanding the properties of gel. Although objective filler characteristics can be measured and identified through rheological data, tests on cohesion properties should also be referenced to predict lifting capacity, migration, moldability, and so on.

This study shows the physical results of fillers from other manufacturers developed with clinically similar indications. This study was analyzed with a limited number of subjects, and it is necessary to analyze experimental data for fillers actually used in the medical field. Multiple repetitive tests would be needed for statistical significance. Further studies are needed to measure the cohesion of different hyaluronic acid fillers and to evaluate the clinical utility of the various characteristics of hyaluronic acid fillers. And also further studies are needed to establish standard cohesion test and range of value.

### Conclusion

We evaluated and compared the fillers of each manufacturer with representative methods known to evaluate cohesion. The results of the tests varied among different hyaluronic acid fillers but each tests showed similar cohesion results. This study identified fillers with different coherence levels than expected, based on their rheological properties (especially complex viscosity result), demonstrating the importance of cohesion testing. Further studies are needed to standardize the cohesion test and importance of cohesiveness at clinical application.

## Important Points

- Cohesion tests yielded varying results for five different hyaluronic acid fillers.
- Hyaluronic acid cohesion test results may provide extra data for plastic surgeons selecting the right filler for patients.
- Rheological data alone may not be sufficient for clinical decision making.
